# Simulated Targeted Anthelmintic Treatment Maintains Dung Removal but Not Dung‐Inhabiting Insect Communities

**DOI:** 10.1002/ece3.74477

**Published:** 2026-10-08

**Authors:** Samuel Appleyard‐Sanderson, Miguel Lurgi, Richard Wall, Christopher F. Clements

**Affiliations:** ^1^ School of Biological Sciences University of Bristol Bristol UK; ^2^ Department of Biosciences Swansea University Swansea UK

**Keywords:** ecosystem, inhabiting insects, livestock management, macrocyclic lactone, modelling, non‐target effects

## Abstract

The intensive use of antiparasitic treatments, such as macrocyclic lactones, is known to affect non‐target dung‐inhabiting insects, leading to reduced community functioning and ecosystem services. We describe the development of a spatially explicit simulation to assess the effects of the treatment of different proportions of a livestock herd with anthelmintics, a strategy known as targeted selective treatment. We simulated the treatment of a varying proportion of a livestock herd with antiparasitics, and the effects inflicted on three dung‐inhabiting insect guilds (flies, endocoprids and paracoprids) with novel life cycle, colonisation and dispersal mechanisms. We varied livestock stocking density to test the effect of dung availability on these effects. Complex population‐level effects were seen as treatment varied, while increased stocking densities led to smaller community sizes overall. Dung removal, measured by total pat number and mean pat biomass, increased with decreasing livestock treatment at all stocking densities. These findings provide a theoretical basis for promoting targeted selective treatment with antiparasitics in an effort to balance livestock production and animal welfare with biodiversity and ecosystem functioning.

## Graphical abstract

Spatially explicit simulation modelling reveals complex effects of anthelmintic treatment regimes on dung‐inhabiting insect communities across three functional guilds (flies, endocoprids, paracoprids). Only without treatment did significant community effects not occur, while increasing livestock stocking density reduced overall community size regardless of treatment level. Dung removal increased as the proportion of treated animals declined across all stocking densities. These findings provide a theoretical basis for targeted selective treatment as a practical strategy to balance livestock production and animal welfare with the conservation of dung insect biodiversity and associated ecosystem services.

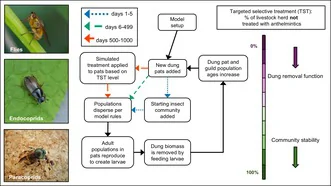

## 1 Introduction

Intensive livestock management practices are considered to be an important contributor to global invertebrate biodiversity declines (Emmerson et al. 2016), particularly affecting dung‐inhabiting insects (Sánchez‐Bayo and Wyckhuys 2019). One procedure that may add to such losses is the intensive use of antiparasitic treatments. Treatments with synthetic endectocides (anthelmintics and insecticides) are used to reduce the rates of infection and infestation of livestock by a wide range of internal and external parasites, including gastrointestinal nematodes, flukes and ectoparasites such as lice, mites and fly larvae. These treatments, therefore, contribute to improved animal welfare and greater livestock productivity (Campbell et al. 1983). However, following application, many of the compounds administered are excreted in faeces or urine in a largely unmetabolised state, where they can have a range of lethal and sub‐lethal effects on non‐target dung‐inhabiting insects (Wall and Beynon 2012).

Amongst the treatments of concern, particular attention has been focused on the macrocyclic lactones, amongst which ivermectin has been the most extensively studied (Jacobs and Scholtz 2015). Dung‐breeding flies and endocoprid beetles experience substantial mortality in dung contaminated with macrocyclic lactone residues for extended periods, particularly during larval development (Floate 1998; O'Hea et al. 2010). Paracoprid beetles, which reproduce and develop primarily in the soil beneath dung pats, have been less extensively studied, but available evidence indicates that both larval and adult stages can also be negatively affected by macrocyclic lactone contamination (Sands and Wall 2018). Sublethal effects, such as reduced fecundity and impaired feeding, have also been documented across flies and beetles (Beynon et al. 2012; Manning et al. 2017; Weaving et al. 2020). The loss of dung‐inhabiting insects may have impacts on nutrient cycling, dung removal and soil architecture, and lead to increases in pest and parasitic gastrointestinal nematode populations in pastures. This risks compromising community functioning and the ecosystem services they provide (Beynon et al. 2012; Sands and Wall 2018). The impacts of macrocyclic lactones extend beyond local dung‐inhabiting insect communities, with cascading effects across the entire pastureland ecosystem, such as reducing prey availability for insectivorous birds and bats, contributing to wider biodiversity declines where endectocides are routinely used (Villar and Schaeffer 2023).

Given the important role of these treatments in animal management, clearly finding an appropriate balance between maintaining production efficiency and animal welfare and the protection of biodiversity and ecosystem functioning is required (Wall et al. 2026). The use of targeted selective treatment (TST) has been proposed as one approach that might help to mitigate the environmental effects of anthelmintic treatment (Wall et al. 2026). Originally proposed as an approach that might reduce the rate of development of resistance in gastrointestinal (GI) parasites, TST involves treating only a proportion of a group of livestock, ideally those with the most clinically significant parasitic loads, thereby leaving a proportion of the herd untreated to provide refugia of susceptible parasites on pasture (Berk et al. 2016). This approach may also support the persistence and recolonisation of insect populations (Cooke et al. 2017; Lewis et al. 2024). However, there are many unknowns, particularly relating to the seasonal and spatial dynamics of insect communities and the effectiveness of this approach remains uncertain.

Modelling has been used previously to explore the effects of endectocides on dung‐inhabiting insect communities (Vale and Grant 2002; Boxall et al. 2007; Cooke et al. 2017). These studies suggest that slower‐breeding species are likely to be the most sensitive and that the impact is likely to increase with treatment dosage and frequency (Vale and Grant 2002; Boxall et al. 2007). However, models have tended to focus on a very limited number of species for which good life‐history data exist, primarily *Scathophaga stercoraria* and short durations typically up to one year (Boxall et al. 2007; Cooke et al. 2017). Furthermore, few models include reported sublethal effects. As a result, the aim of the work described here was to develop a spatially explicit simulation to assess the effects of the treatment with anthelmintic of different proportions of a herd and at different stocking densities, on representative guilds within a dung‐inhabiting insect community in a temperate pasture ecosystem.

## 2 Methods

### 2.1 Model Overview

The model included three guilds of dung‐inhabiting insects typically found in temperate pasture ecosystems: flies, endocoprid beetles and paracoprid beetles. These guilds were chosen to capture functional diversity while allowing aggregation of species‐level life history data. Guild‐specific life histories, colonisation and dispersal and dung degradation were simulated over 1000 timesteps, while anthelmintic exposure and dung availability were manipulated to test their effects on the community. Predatory and parasitic species were not considered to allow consumer‐resource interactions to be isolated and to simplify the model.

Dung‐breeding flies were parametrised using life history data from several coprophagous dipterans (notably *Sepsidae* spp. and *Scathophaga stercoraria* ) and characterised as having high fecundity and fast development with low per capita dung removal and a high sensitivity to anthelmintics at all life stages (Blanckenhorn 1997; Blanckenhorn et al. 2013; Mann et al. 2015). Conversely, the endocoprid and paracoprid beetle guilds were parametrised using life history data from predominantly *Aphodius, Onthophagus* and *Geotrupes* spp., and the guilds were characterised by slower development, lower fecundity and higher dung removal rates. Adult beetles experienced low to medium sensitivity to anthelmintics in contaminated pats, but larvae experienced high mortality and sensitivity to sublethal effects (O'Hea et al. 2010; Sands and Wall 2018; Tonelli et al. 2020). All life history parameters and their sources from the literature are presented in Table 1.

**TABLE 1:** Key biological parameters relevant to the population and functional dynamics of dung‐inhabiting guilds present in the model.

Parameter	Guild	Value	Source
Egg‐to‐adult development (days)	1	20	1—*Sepsis* spp.
	2	45	5—*Aphodius* spp., 15— *Aphodius fimetarius*
	3	100	4— *Onthophagus nuchicornis* , 5
Proportion of time spent in larval stage	1	0.5	Author estimate due to no published parameter found
	2	0.75	5, 6—*Aphodius* spp.
	3	0.75	5, 6—*Aphodius* spp.
Fecundity (no. of eggs)	1	95	2—*Scathophaga stercoraria, Sepsis cynipsea*, 12—*Lucilia sericata*
	2	8	5, 6—*Aphodius* spp.
	3	15	7— *O. nuchicornis* , 10—*Geotrupes spiniger, Trypocopris (Geotrupes) vernalis*
Inter‐clutch period (days)	1	8	2—*S. stercoraria, S. cynipsea*
	2	40	6—*Aphodius* spp.
	3	260	9— *Onthophagus binodis*
Life expectancy (days)	1	24	2—*S. stercoraria, S. cynipsea*
	2	40	5—*Aphodius* spp., 14— *A. fimetarius*
	3	260	9— *O. binodis*
Dung removal (g/day/individual)	1	0.0055	8— *S. stercoraria*
	2	0.08	12—*Aphodius fossor*, 17—*Aphodius* spp.
	3	0.455	17—*Onthophagus* spp.
Adult mortality (%)	1	1	3—*Sepsis* spp., 15— *S. stercoraria*
	2	0.2	Author estimate from 13—*Aphodius ater, A* *. rufipes*
	3	0.2	Author estimate from 16—*Onthophagus* spp., *Aphodius* spp.

*Note:* For all parameters, values were means used in a normal distribution (standard deviation = mean ×0.1) to estimate a set of parameters for each repeated simulation.

*Source:* 1—Blanckenhorn et al. (2013), 2—Blanckenhorn (1997), 3—Floate (1998), 4—Floate et al. (2015), 5—Gittings (1994), 6—Gittings and Giller (1997), 7—Indigo et al. (2025), 8—Kavanaugh and Manning (2020), 9—Kotiaho and Simmons (2003), 10—Kühne (1996), 11—Mann et al. (2015), 12—Manning et al. (2017), 13—O'Hea et al. (2010), 14—Pokhrel et al. (2020), 15—Römbke et al. (2009), 16—Sands and Wall (2018), 17—Tonelli et al. (2020).

### 2.2 Spatial and Field Structure

A pasture was modelled as a 20 by 20 grid of 400 square cells each capable of holding a single dung pat (represented in Figure 1a). To simulate the sizes of naturally voided pats, each dung pat received a biomass drawn from a normal distribution, $Ν\left(1025,102.5\right)$, upon initialisation which was allowed to decay naturally or by insects. Dung pat wet weights observed in the field typically vary substantially, and here they were allowed to range from approximately 550 g to 1.5 kg.

**FIGURE 1:**
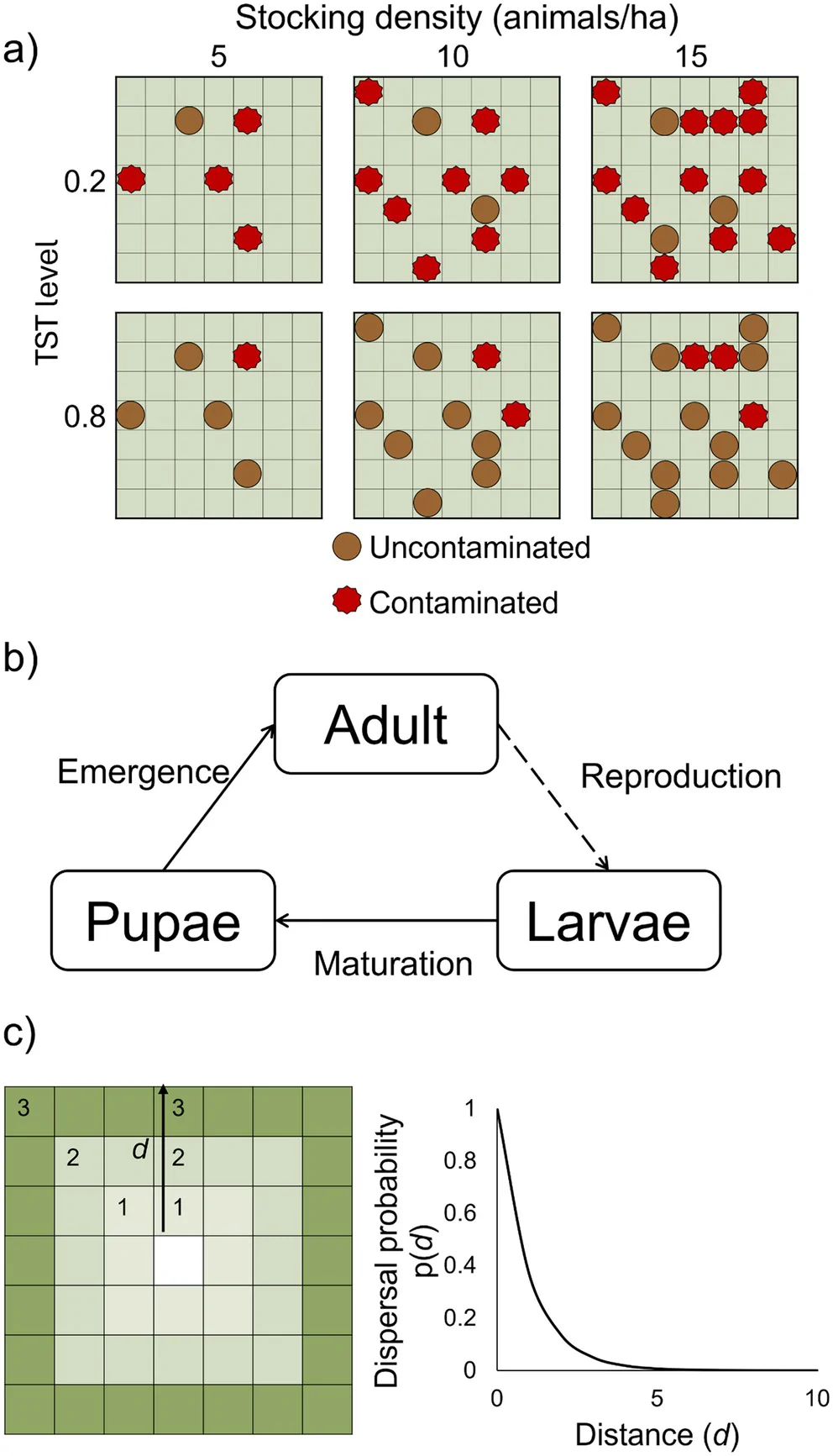
Schematic representations of key model procedures. (a) A spatial representation of the two independent variables (targeted selective treatment level and stocking density) used in the model. Red, pointed circles represent contaminated pats; brown smooth circles represent uncontaminated pats. (b) The life cycle followed by all individuals in the model. Solid lines represent the 1:1 transfer of individuals, dotted lines represent the created of individuals. (c) A visual representation of the connectivity and dispersal mechanism used in the model. The distance, *d*, of a destination cell from the dispersing cell dictates the probability of an individual dispersing to the destination cell.

Each cell had an equal chance of receiving a pat, and the number of pats generated per day was stipulated by stocking densities of 5, 10 and 15 animals/ha. Each cell held four distinct populations, representing three life stages for each guild: larvae, pupae, adults in the dung pat and adults in the field. Adults in the field were able to disperse to other cells each time step, governed by the connectivity value between source and destination cells.

Connectivity values between cells were determined by a spatial connectivity matrix C for the pasture, a square lattice of $N={\text{size}}^{2}$ cells, using a process modified from Chen et al. (2022) (Figure 1c). The distance d between cells with dispersing populations i and potential cell destinations, j, with coordinates $\left(x_{i},y_{i}\right)$ and $\left(x_{j},y_{j}\right)$ respectively, was defined as the Chebyshev distance as shown in Equation (1) (Cantrell 2000):

(1) $$d\left(i,j\right)=\max\left(\left|x_{i}-x_{j}\right|,\left|y_{i}-y_{j}\right|\right)$$

Dispersal probabilities from a focal cell were then calculated from an exponentially decaying function of the distance d from that cell as shown in Equation (2):

(2) $$p\left(d\right)=\frac{1-F\left(d;\lambda \right)}{\sum_{r=1}^{\text{size}-1}\left(1-F\left(\mathrm{r\lambda}\right)\right)}$$

where $F\left(\mathrm{d\lambda}\right)=1-e^{-\mathrm{\lambda d}}$, was an exponential cumulative distribution function with rate λ. The connectivity matrix was then defined as shown in Equation (3):

(3) $$C_{\mathrm{ij}}=\left\{\begin{array}{c} 0,\\ p\left(d\left(i,j\right)\right), \end{array}\begin{array}{c} i=j,\\ i\neq j, \end{array}\right.$$

and normalised such that $\sum_{j}C_{\mathrm{ij}}=1$ for all i.

### 2.3 Insect Life Cycle

Individuals developed through three life stages in the model (Figure 1b). Egg‐to‐adult development was split into a larval and a pupal stage, distinguished for flies and endocoprids by larval, but not pupal, dependence on dung biomass for survival. Larvae of paracoprid beetles, which remove dung from the pat immediately and bury it below in the soil‐dung layer, did not depend on dung biomass for survival (Beynon et al. 2012). It was assumed that the time spent as eggs before hatching was negligible and was not explicitly modelled. Larval population sizes were calculated as the product of the adult population size and guild‐specific fecundity divided by two to simulate the contribution of only females to population growth; a 1:1 sex ratio was assumed throughout. Larval and pupal durations were defined by guild‐specific development periods (Table 1), after which adults entered the field population.

In contaminated pats, larval populations experienced 100% lethality. Despite evidence of altered development rates, changes in development rate in contaminated pats were not incorporated in the current model (O'Hea et al. 2010; Blanckenhorn et al. 2013). Natural background mortality was applied to all adult populations, regardless of location, every time step, calculated as the inverse of guild life expectancy in uncontaminated pats (Table 1).

### 2.4 Dispersal and Colonisation

The initial field community in a cell, added to the model for the first five days (Figure 2), was determined using a total community abundance drawn from a normal distribution, $Ν\left(500,50\right)$. This abundance was then split into an expected typical guild structure of 90% flies, 5% endocoprid beetles and 5% paracoprid beetles (Lee and Wall 2006a). Pat colonisation occurred when adults were moved from the field population of a cell to the pat population in a guild‐specific time‐dependent colonisation pattern: flies were allowed to colonise only one‐day‐old pats, while beetles colonised pats for up to 5 days after deposition, to reflect known broad successional colonisation patterns (Floate et al. 2005). On colonising a contaminated pat, fly adults suffered 100% mortality; however, adult beetle populations suffered variable mortality after colonisation to reflect the known differences in adult beetle sensitivity (Table 1). Colonising insects were assumed to exhibit neither attraction nor repulsion to contaminated pats, despite evidence of both behaviours (Finch et al. 2020).

**FIGURE 2:**
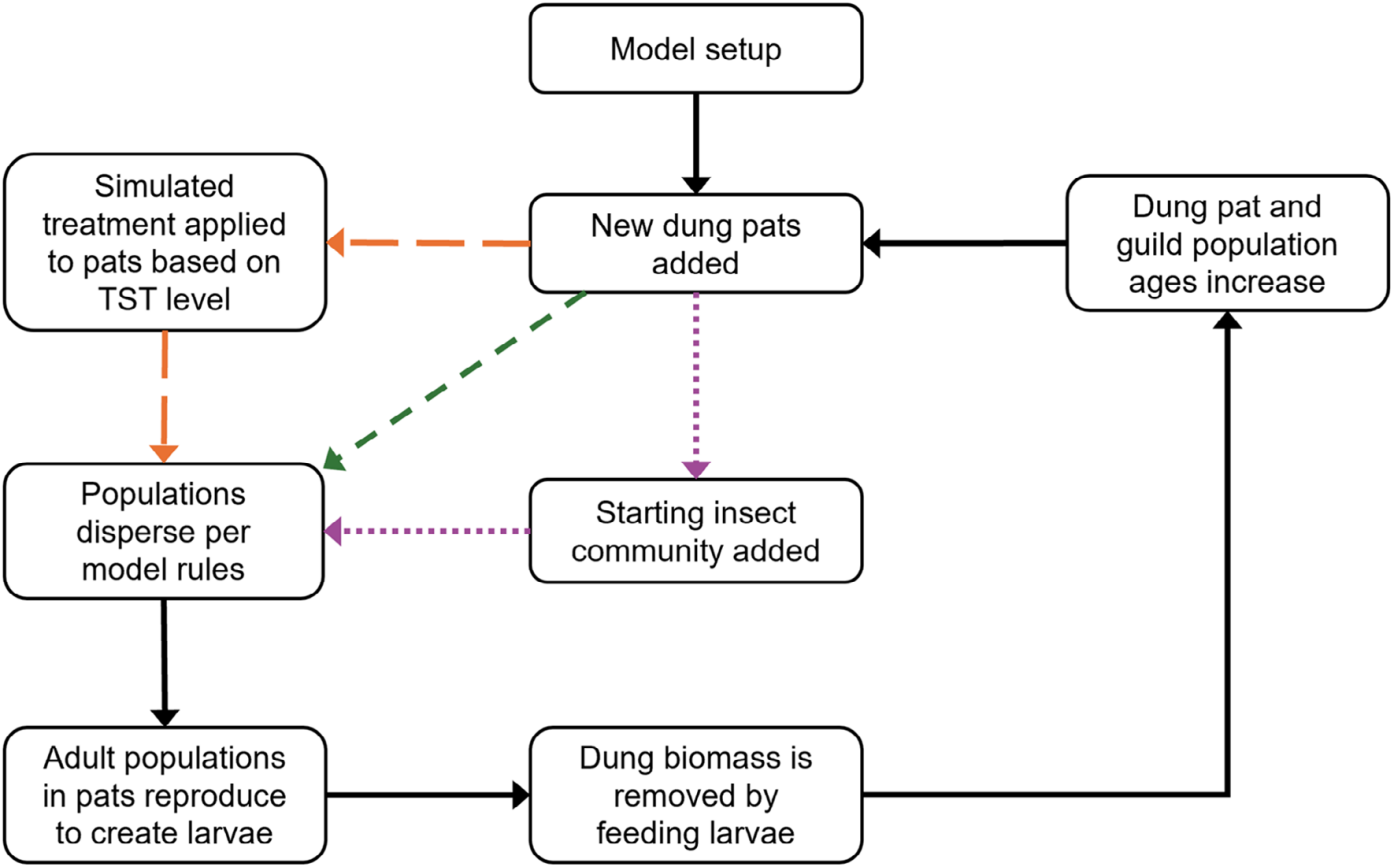
Workflow diagram of the simulation model. Arrows with different colours and line types represent processes conditional on simulation day: Black arrows occur every time step (day), purple and thin dashes indicate actions taken during model initialisation (days < 6), green and medium dashes indicate actions before the contamination begins (5 < days < 500), orange and thick dashes indicate actions during the contamination phase (days > 500).

Dispersal was modelled as a multinomial distribution of dispersing adult populations amongst cell destinations, based on the cell‐specific connectivity values, $C_{\mathrm{ij}}$ (Equation 3). The probability of dispersal from cell i to cell j was proportional to $C_{\mathrm{ij}}$, the connectivity between the two cells. Movement was thus distance‐dependent and allowed between patches in all eight directions, that is, a Moore neighbourhood (Raol 2016) (Figure 1c).

### 2.5 Dung Degradation

Dung biomass $\left(B_{t}\right)$ decayed via two processes: (1) natural background decay $\left(D_{\mathrm{nat}}\right)$ driven by climate process and soil microbial community (Lee and Wall 2006b) and (2) degradation facilitated by the coprophagous community present $\left(D_{\mathrm{ins}}\right)$. $D_{\mathrm{nat}}$ was modelled, assuming a typical temperate spring–summer climate, as a stochastic loss of biomass (g) drawn from a normal distribution ($Ν\left(5,0.5\right)$); a loss of 5 g a day would cause a pat to fully degrade in approximately six months. $D_{\mathrm{ins}}$ was assumed to occur only by the action of the larval population and modelled as the sum of larval per capita dung removal across all guilds as shown in Equation (4):

(4) $$D_{\mathrm{ins}}=\sum_{g}r_{g,\text{treat}}L_{g,t}$$

where $r_{g,\text{treat}}$ is the guild‐ and treatment‐specific dung removal rate ($g\;{\text{larva}}^{-1}{\mathrm{day}}^{-1}$), and $L_{g,t}$ is the larval abundance in a pat. Dung removal by coprophagous insects was reduced by 50% in contaminated pats as a sublethal effect in the model (Jargalsaikhan et al. 2023). Dung biomass decay is therefore modelled as shown in Equation (5):

(5) $$B_{t+1}=\max\left(0,B_{t}-D_{\mathrm{nat}}-D_{\mathrm{ins}}\right)$$

ensuring biomass could not fall below zero.

Total pat decay caused fly and endocoprid larval populations to die and all adult populations to disperse. All pupae and paracoprid larvae were unaffected, as they were considered to be no longer dependent on the dung pat at this stage. Pat contamination was assumed not to affect dung degradation in the absence of insects (Floate et al. 2005).

### 2.6 Anthelmintic Treatment

For the purpose of parametrisation, simulated contamination of pats with antiparasitics was assumed to be based on a subcutaneous injection of a herd with ivermectin at 200 $\mathrm{\mu g}\;{\mathrm{kg}}^{-1}$ BW, reflecting a common method of application at the recommended dose, included in previous literature studying the effect of ivermectin on dung‐inhabiting insects (summarised in table 1 of Urrutia et al. 2024). The proportion of animals treated, and thus pats contaminated, was varied according to the selected TST level. Each newly generated pat was assigned a contamination state drawn from a Bernoulli trial where the probability of a dung pat being contaminated with an anthelmintic was determined by the TST level. Here, TST level described the proportion of untreated animals, such that a TST of 0 meant that all animals were treated and a TST of 1 meant that no animals were treated. Degradation of macrocyclic lactone concentrations after excretion has been recorded in both dung and soil (O'Hea et al. 2010). However, here the degradation of residues was not considered, and the pats remained fully contaminated until they degraded.

### 2.7 Simulation Procedure

Model simulations comprised 1000 steps, representing daily changes over a period of approximately five continuous grazing seasons of approximately 6 months each (summarised in Figure 2). An initialisation period occurred at the start of each simulation where communities of a set size populated the first pats generated in the model. Treatment initiated at day 500, with newly deposited pats classified as contaminated or uncontaminated. 10 replicate simulations were run for each combination of stocking densities (5, 10 and 15 animals/ha) and TST levels (0 to 1, increasing in increments of 0.1), totalling 330 individual runs. Simulations were implemented in R v4.5.2 (R Core Team 2025), with parallelisation via *foreach* (Microsoft and Weston 2022a) and *doParallel* (Microsoft and Weston 2022b).

### 2.8 Data Analysis

All simulation results reported are the adult populations in dung pats. Model outputs were filtered to exclude the initial 500‐day stabilisation period and averaged across all replicate simulations. The abundances for each guild were then divided by the number of pats at each day and TST level to give the density. The mean population density and the 25th, and 75th percentiles were then calculated for each TST level. Pat biomass values were tracked within the model and then averaged across replicate simulations. The mean, 25th, and 75th percentiles for pat biomass were then calculated for each TST level. R packages *dplyr*, *tidyr*, *stringr* and *ggplot2* were used for analysis and visualisation (Wickham 2016, 2025; Wickham et al. 2023, 2026).

## 3 Results

### 3.1 Effects on Insect Density

Mean insect community density decreased slightly as TST increased from 0 to 0.4 but then began increasing exponentially as TST increased to 1 (Figure 3). This was caused by the change in community structure and the population density of each guild. Fly population density increased rapidly and consistently with TST level; flies had the smallest population density of the guilds at TST = 0 and the largest at TST = 1. Endocoprid beetle population density increased at a slower rate, and paracoprid beetle population density decreased exponentially, but at a slow rate.

**FIGURE 3:**
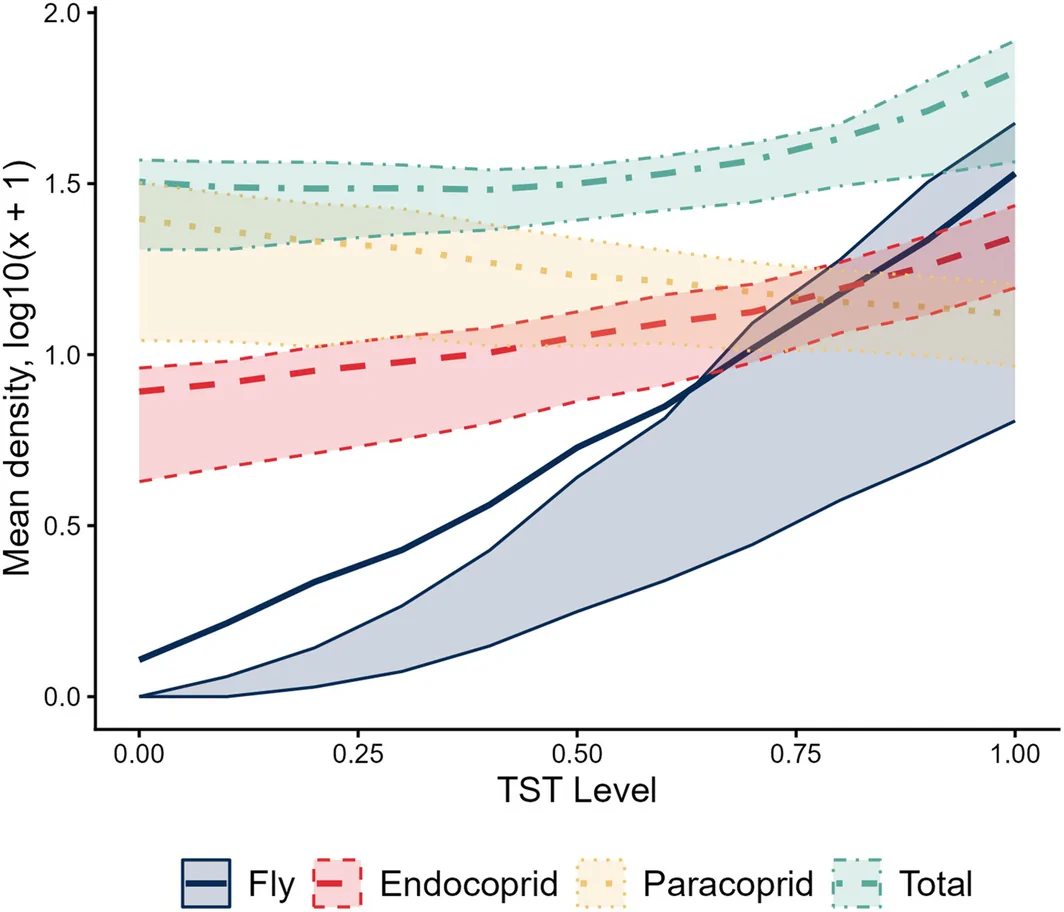
Mean population density of guilds (fly—solid navy, endocoprid—red dashed, paracoprid—yellow dotted) and the total community density (green dot‐dashed) across TST levels 0–1. Mean density was calculated as the population abundance per dung pat. The corresponding shaded areas show the 25th and 75th percentiles for each plotted mean density.

Figures 3 and 5 report the response variables of community and population densities, calculated as abundances per pat and so it is important to report how pat numbers are affected by both TST level and stocking density. Increasing TST level substantially reduced total pat numbers, particularly towards the end of the model run (days 800–1000) (Figure 4). Stocking density had the largest effect on total pat number at TST = 0 and decreased with increasing TST level, with almost no effect when all pats were uncontaminated (TST = 1).

**FIGURE 4:**
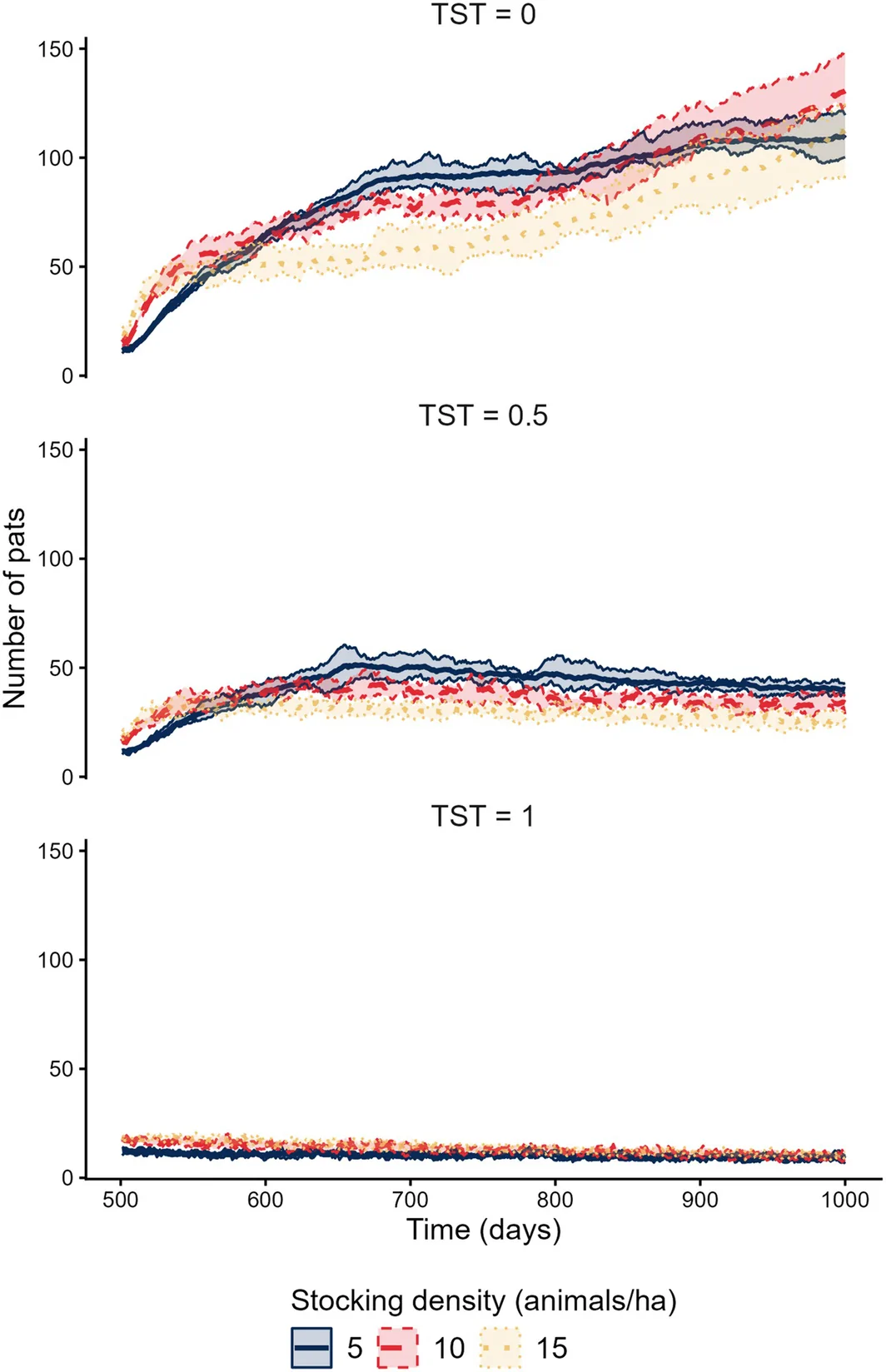
Mean number of pats at the lowest (0), middle (0.5) and highest (1) TST levels, and at the three stocking densities across the contamination phase (days 500–1000). The corresponding shaded areas show the 25th and 75th percentiles for each plotted mean number of pats.

Stocking density changed the effect of TST level on guild population density substantially. At 5 animals/ha, numbers increased exponentially to the maximum seen across the TST levels (Figure 5). At the medium stocking density of 10 animals/ha, insect numbers decreased slightly up to TST = 0.5 but then increased exponentially as the TST level approached 1. At the highest stocking density of 15 animals/ha, the numbers of insects were highest at TST = 0, decreased as TST level changed from 0 to 0.75 but then increased again slightly as TST level approached 1 (Figure 5).

**FIGURE 5:**
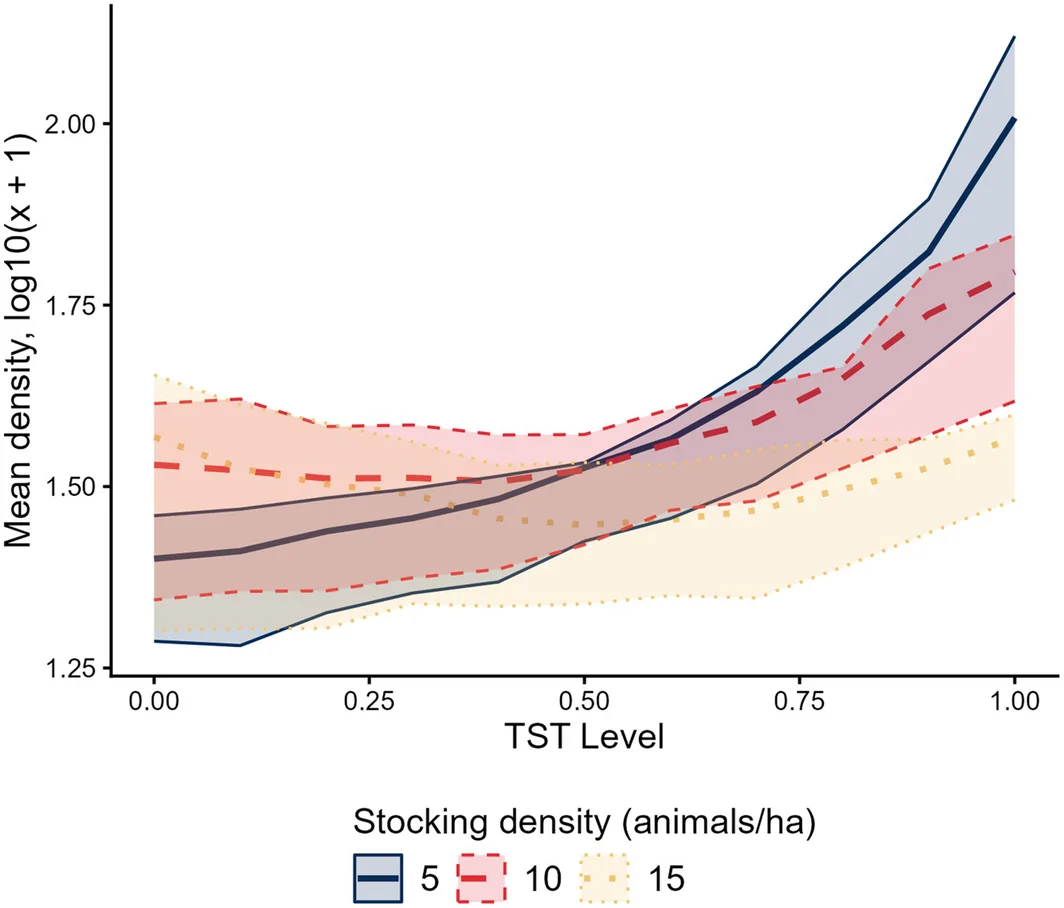
Mean community density across TST levels 0–1 split by stocking density (5 animals/ha—navy solid, 10 animals/ha—red dashed, 15 animals/ha—yellow dotted). Mean density was calculated as the population abundance per dung pat. The corresponding shaded areas show the 25th and 75th percentiles for each plotted mean density.

### 3.2 Effect on Standing Pat Biomass

Mean standing dung pat biomass decreased exponentially as the TST level increased (Figure 6); however, the rate of change depended on the stocking density. At the lowest density (5 animals/ha), and thus lowest dung availability, biomass was reduced by around 13%. This increased to 20% and 31% for medium (10 animals/ha) and high (15 animals/ha) stocking densities, respectively. At all stocking densities, the decrease changed exponentially beyond TST = 0.5, while the decrease from TST = 0 to 0.5 was more linear.

**FIGURE 6:**
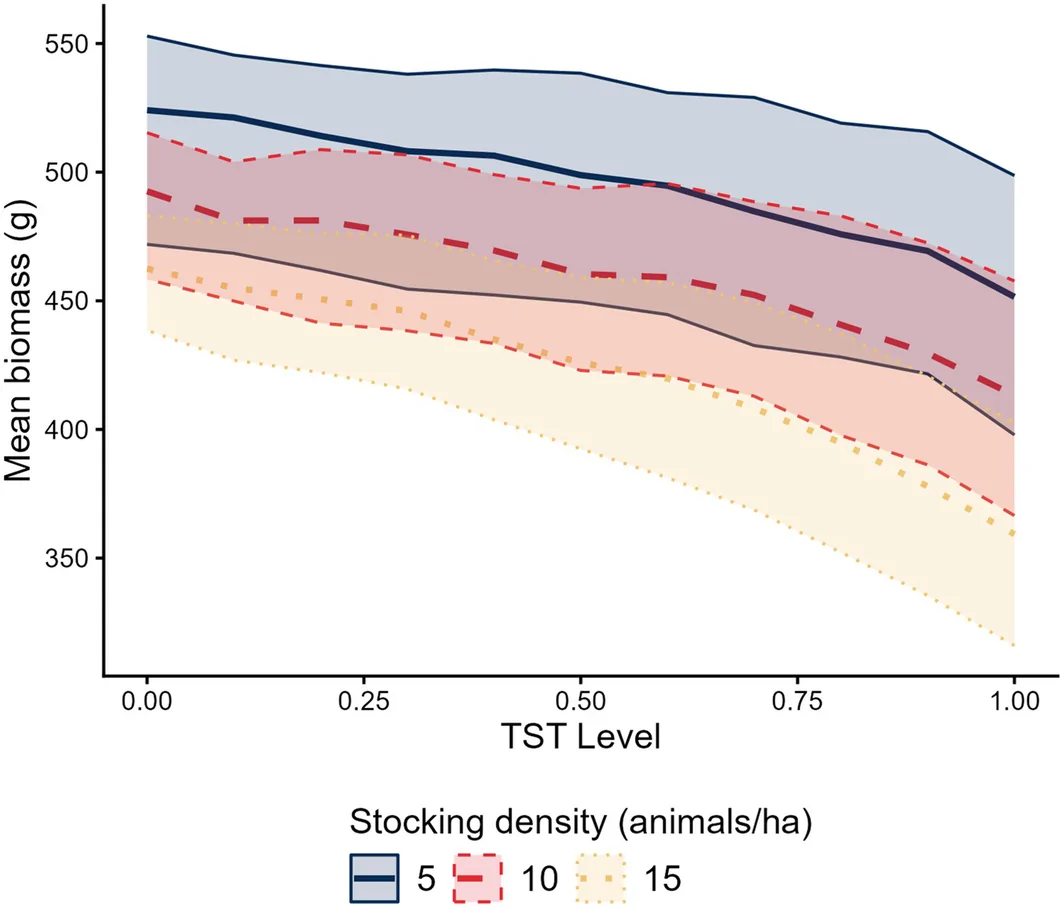
Mean pat biomass (g) across TST levels 0–1 split by stocking density (5 animals/ha—navy solid, 10 animals/ha—red dashed, 15 animals/ha—yellow dotted). The corresponding shaded areas show the 25th and 75th percentiles for each plotted mean biomass.

## 4 Discussion

In this study, a simulation model has been used to explore the impacts of targeted selective treatment (TST) and stocking density on the effects of anthelmintic treatment on non‐target dung‐inhabiting insects. The simulations suggest that the use of TST will allow a higher proportion of dung‐inhabiting insects to persist; however, population‐level effects may be complex. All TST level and stocking density combinations resulted in substantial changes to community structure because of the differential effects of treatment on the various guilds included. The complex outcomes were highlighted by the observation that, here, the numbers of adult paracoprid beetles decreased as the proportion of animals left untreated increased. An explanation for this is that the paracoprid guild used in the model included such a wide diversity of species. The variation in life history trait distributions calculated may then have resulted in overgeneralised parameters. Greater accuracy may have resulted from the use of more fine‐grained categories. Previous work has identified paracoprid populations as consistently smaller in size than flies, and so, at TST = 1, community structure could be described as returning to a more natural state (Lee and Wall 2006a). Such complex community‐level changes were identified by Sands and Wall (2018), where substantial landscape changes in beetle community structure were associated with long‐term use of different classes of anthelmintics and insecticides. Previous work has also concluded that a TST approach is likely to help maintain insect populations, which we build upon here (Cooke et al. 2017; Lewis et al. 2024).

Little previous work has explored the relationships between stocking density, antiparasitic use and insect populations. Higher stocking densities were found to be associated with smaller insect populations, largely because farms employing higher stocking densities were more likely to use more intensive husbandry practices (Hutton and Giller 2003). Here, higher stocking density was associated with a smaller average community size. It seems likely that in the model, the increased available dung biomass resulted initially in higher population densities, which led to rapid pat degradation and an overcompensatory density‐dependent competitive exclusion of some populations, which in turn drove an overall decrease over the timescale studied. This result could perhaps model natural competition, although the role of competition in this dung community system dynamics has been given little attention and further research into its impact, particularly in conjunction with the use of TST, is required. Livestock were modelled as remaining on a single pasture for the entire simulation period, with no break in dung pat input after the start of a simulation, in order to maintain model simplicity. The densities modelled here should be interpreted as representing the upper end of UK grazing intensity, and results at the higher stocking densities (10–15 animals/ha) may be more directly applicable to intensively or rotationally managed systems than to the continuous stocking typical of extensive pasture. Further development of the model, alongside parameterisable data on the effect of anthelmintics on insects at specific stocking densities, would yield more accurate results for these variables.

Both the total number of pats and mean pat decreased as a greater proportion of animals were untreated at all stocking densities. This suggests that dung removal increased, likely due to the lower impact of anthelmintic treatment on larval populations. There have been no empirical field studies of the impact of TST on dung‐inhabiting insects or rates of dung removal. Nevertheless, a 48.6% reduction in the rate of dung removal in pats contaminated with ivermectin was recorded in mesocosms (Manning et al. 2017) and a 32% reduction when both beetle and fly species are present (Kavanaugh and Manning 2020). Notably, higher stocking densities led to a lower average dung biomass at all TST levels. This is likely to have been due to the increased dung biomass generated at the beginning of the simulation, allowing a larger community to develop and then resulting in reduced dung biomass levels, despite the higher rate of dung pat generation at higher stocking densities.

Modelling dung‐community dynamics is problematic because of the lack of data for a wide range of life history parameters, particular fecundity and dispersal. As a result, numerous assumptions and simplifications were made here. The modelled community included only three guilds: flies, endocoprid beetles and paracoprid beetles, resulting in a greatly simplified system losing the complexity of species seasonality and interaction. Behavioural and demographic processes were also simplified. Future models could include a broader diversity of function guilds, including predatory and parasitic species; however, this would require substantial data availability for parametrisation. Furthermore, an explicit mechanism of density dependence that increases larval, pupal and emerging adult mortality based on population size already in a single dung pat could be included to explore this effect and the impact of density dependence on a multi‐guild community. Further research, specifically empirical testing of targeted selective treatment on a multi‐guild community, is required at a multi‐farm scale to allow for the inclusion of species‐specific dispersal in future models.

In the model developed, the guild‐level colonisation rules, where flies were only allowed to colonise pats for 1 day after deposition and beetles were allowed to colonise pats for up to 5 days, may have prevented overlapping colonisation waves that are observed in field systems (Tixier et al. 2015). This was excluded for model simplicity, and dispersal was simplified as distance‐dependent, excluding long‐range recolonisation and landscape connectivity. Furthermore, seasonal dynamics were omitted, representing continuous summer conditions. Consequently, the model likely overestimated year‐round persistence relative to temperate systems that experience overwintering mortality and variable dung availability. Future modelling efforts could explore the effect of seasonal dynamics more closely, as specific impacts of temperate and precipitation on dung beetle communities are known (Krüger and Scholtz 1998a, 1998b; Gittings and Giller 1999). Moreover, although studies have examined the distinction between successive ‘waves’ of colonisation by different species, this phenomenon warrants further investigation in northern temperate environments to provide empirical evidence for incorporation into future models, thereby improving the accuracy of colonisation dynamics (Tixier et al. 2015).

Here, macrocyclic lactone concentrations in dung were held constant at 100% in voided dung until it was decomposed, allowing maximum lethal and sublethal effects throughout the simulation. In reality, residues may decline over time and this may, therefore, have exaggerated long‐term mortality. It would be advantageous in future studies to simulate progressively deteriorating residue concentrations, although while this may change the magnitude or speed of the effects seen, it would not be expected to change the nature of the conclusions. In addition, lethal and sublethal effects were simplified or excluded. For example, mortality in adult Diptera was considered to be 100% in contaminated pats. This reflects consistent high rates of mortality recorded in studies in Sepsidae sp. and *S. stercoraria* . Adult beetle mortality was assumed to vary between 0% and 40% based on mixed empirical findings between species (O'Hea et al. 2010; Sands and Wall 2018). Such effects should be included in a more empirically accurate model.

Our model suggests that reducing the proportion of a livestock herd treated with antiparasitic to any more than 50% had comparatively little further effect on beetle populations, although this apparent threshold is likely to be sensitive to the specific parameter values used, several of which remain poorly constrained empirically and should therefore be treated as indicative rather than as a precise management target. In contrast, fly populations continued to show a clear positive response to reduced treatment beyond this level; flies are, however, the guild with the lowest relative contribution to ecosystem functioning (Beynon et al. 2015). Lower stocking densities appear beneficial for communities despite fewer micro‐habitats, particularly when paired with increasing TST level. However, increasing both TST level and stocking yields the largest effect on dung removal, reducing the total number of pats and mean pat biomass. These findings have direct relevance for livestock management and agri‐environment policy, providing a theoretical basis for promoting targeted‐selective treatment as a compromise between parasite control and biodiversity conservation within precision livestock systems.

## Author Contributions

**Samuel Appleyard‐Sanderson:** methodology (lead), writing – original draft (lead), writing – review and editing (lead). **Miguel Lurgi:** methodology (equal), writing – original draft (equal), writing – review and editing (equal). **Richard Wall:** conceptualization (lead), funding acquisition (lead), writing – original draft (equal), writing – review and editing (equal). **Christopher F. Clements:** methodology (lead), project administration (lead), supervision (lead), writing – original draft (equal), writing – review and editing (equal).

## Funding

Boehringer Ingelheim, Bristol Centre for Agricultural Innovation (Grant S100771‐101).

## Conflicts of Interest

The authors declare no conflicts of interest.

## Data Availability

Data sharing is not applicable to this article as no datasets were generated and all data used in this paper are derived from published literature cited in the text. All simulation code used in this study is available at Zenodo: https://doi.org/10.5281/zenodo.19402244.

## References

[ece374477-bib-0001] Berk, Z. , Y. C. S. M. Laurenson , A. B. Forbes , and I. Kyriazakis . 2016. “Modelling the Consequences of Targeted Selective Treatment Strategies on Performance and Emergence of Anthelmintic Resistance Amongst Grazing Calves.” International Journal for Parasitology: Drugs and Drug Resistance 6, no. 3: 258–271. 10.1016/j.ijpddr.2016.11.002. PMC5137182 27915061

[ece374477-bib-0002] Beynon, S. A. , D. J. Mann , E. M. Slade , and O. T. Lewis . 2012. “Species‐Rich Dung Beetle Communities Buffer Ecosystem Services in Perturbed Agro‐Ecosystems.” Journal of Applied Ecology 49, no. 6: 1365–1372. 10.1111/j.1365-2664.2012.02210.x.

[ece374477-bib-0003] Beynon, S. A. , W. Wainwright , and W. Christie . 2015. “The Application of an Ecosystem Services Framework to Estimate the Economic Value of Dung Beetles to the U.K. Cattle Industry.” Ecological Entomology 40, no. Suppl. 1: 124–135. 10.1111/een.12240.

[ece374477-bib-0005] Blanckenhorn, W. U. 1997. “Altitudinal Life History Variation in the Dung Flies *Scathophaga stercoraria* and *Sepsis cynipsea* .” Oecologia 109, no. 3: 342–352. 10.1007/s004420050092. 28307530

[ece374477-bib-0006] Blanckenhorn, W. U. , N. Puniamoorthy , M. A. Schäfer , A. Scheffczyk , and J. Römbke . 2013. “Standardized Laboratory Tests With 21 Species of Temperate and Tropical Sepsid Flies Confirm Their Suitability as Bioassays of Pharmaceutical Residues (Ivermectin) in Cattle Dung.” Ecotoxicology and Environmental Safety 89: 21–28. 10.1016/j.ecoenv.2012.10.020. 23260241

[ece374477-bib-0007] Boxall, A. B. A. , T. N. Sherratt , V. Pudner , and L. J. Pope . 2007. “A Screening Level Index for Assessing the Impacts of Veterinary Medicines on Dung Flies.” Environmental Science and Technology 41, no. 7: 2630–2635. 10.1021/es0618705. 17438826

[ece374477-bib-0008] Campbell, W. C. , M. H. Fisher , E. O. Stapley , G. Albers‐Schönberg , and T. A. Jacob . 1983. “Ivermectin: A Potent New Antiparasitic Agent.” Science 221, no. 4613: 823–828. 10.1126/science.6308762. 6308762

[ece374477-bib-0009] Cantrell, C. D. 2000. Modern Mathematical Methods for Physicists and Engineers. Cambridge University Press.

[ece374477-bib-0010] Chen, S. , J. Shi , Z. Shuai , and Y. Wu . 2022. “Global Dynamics of a Lotka–Volterra Competition Patch Model.” Nonlinearity 35, no. 2: 817–842. 10.1088/1361-6544/ac3c2e.

[ece374477-bib-0011] Cooke, A. S. , E. R. Morgan , and J. A. J. Dungait . 2017. “Modelling the Impact of Targeted Anthelmintic Treatment of Cattle on Dung Fauna.” Environmental Toxicology and Pharmacology 55: 94–98. 10.1016/j.etap.2017.07.012. PMC5637707 28843101

[ece374477-bib-0012] Emmerson, M. , M. B. Morales , J. J. Oñate , et al. 2016. “How Agricultural Intensification Affects Biodiversity and Ecosystem Services.” In Advances in Ecological Research, edited by A. J. Dumbrell , R. L. Kordas , and G. Woodward , vol. 55, 43–97. 10.1016/bs.aecr.2016.08.005.

[ece374477-bib-0013] Finch, D. , H. Schofield , K. D. Floate , L. M. Kubasiewicz , and F. Mathews . 2020. “Implications of Endectocide Residues on the Survival of Aphodiine Dung Beetles: A Meta‐Analysis.” Environmental Toxicology and Chemistry 39, no. 4: 863–872. 10.1002/etc.4671. 32181912

[ece374477-bib-0014] Floate, K. D. 1998. “Off‐Target Effects of Ivermectin on Insects and on Dung Degradation in Southern Alberta, Canada.” Bulletin of Entomological Research 88, no. 1: 25–35. 10.1017/s0007485300041523.

[ece374477-bib-0015] Floate, K. D. , K. G. Wardhaugh , A. B. A. Boxall , and T. N. Sherratt . 2005. “Fecal Residues of Veterinary Parasiticides: Nontarget Effects in the Pasture Environment.” Annual Review of Entomology 50, no. 1: 153–179. 10.1146/annurev.ento.50.071803.130341. 15471531

[ece374477-bib-0016] Floate, K. D. , D. W. Watson , P. Coghlin , and O. Olfert . 2015. “Degree‐Day Models for Development of the Dung Beetles *Onthophagus nuchicornis* , *O. taurus*, and *Digitonthophagus gazella* (Coleoptera: Scarabaeidae), and the Likelihood of *O. taurus* Establishment in Southern Alberta, Canada.” Canadian Entomologist 147, no. 5: 617–627. 10.4039/tce.2014.70.

[ece374477-bib-0017] Gittings, T. 1994. The Community Ecology of Aphodius Dung Beetles. PhD thesis, National University of Ireland. 10.13140/RG.2.2.23796.55685.

[ece374477-bib-0018] Gittings, T. , and P. Giller . 1999. “Larval Dynamics in an Assemblage of *Aphodius* Dung Beetles.” Pedobiologia 43, no. 5: 439–452. 10.1016/S0031-4056(24)00566-3.

[ece374477-bib-0019] Gittings, T. , and P. S. Giller . 1997. “Life History Traits and Resource Utilisation in an Assemblage of North Temperate *Aphodius* Dung Beetles (Coleoptera: Scarabaeidae).” Ecography 20, no. 1: 55–66. 10.1111/j.1600-0587.1997.tb00347.x.

[ece374477-bib-0020] Hutton, S. A. , and P. S. Giller . 2003. “The Effects of the Intensification of Agriculture on Northern Temperate Dung Beetle Communities.” Journal of Applied Ecology 40, no. 6: 994–1007. 10.1111/j.1365-2664.2003.00863.x.

[ece374477-bib-0021] Indigo, A. , K. Stokes , O. Burchell , and P. Manning . 2025. “The Non‐Indigenous Dung Beetle ( *Onthophagus nuchicornis* ) Can Effectively Reproduce Using the Dung of Indigenous Eastern North American Mammals.” PeerJ 13: e18674. 10.7717/peerj.18674. PMC11702356 39763702

[ece374477-bib-0022] Jacobs, C. T. , and C. H. Scholtz . 2015. “A Review on the Effect of Macrocyclic Lactones on Dung‐Dwelling Insects: Toxicity of Macrocyclic Lactones to Dung Beetles.” Onderstepoort Journal of Veterinary Research 82, no. 1: a858. 10.4102/ojvr.v82i1.858. PMC6238710 26017637

[ece374477-bib-0023] Jargalsaikhan, P. , G. Altangerel , T. Enkhchimeg , U. Aibek , and B. Bayartogtokh . 2023. “Variation in Dung Removal Rates by Dung Beetles (Coleoptera: Scarabaeoidea) in a Temperate, Dry Steppe Ecosystem.” Diversity 15, no. 1: 91. 10.3390/d15010091.

[ece374477-bib-0024] Kavanaugh, B. , and P. Manning . 2020. “Ivermectin Residues in Cattle Dung Impair Insect‐Mediated Dung Removal but Not Organic Matter Decomposition.” Ecological Entomology 45, no. 3: 671–678. 10.1111/een.12838.

[ece374477-bib-0025] Kotiaho, J. S. , and L. W. Simmons . 2003. “Longevity Cost of Reproduction for Males but no Longevity Cost of Mating or Courtship for Females in the Male‐Dimorphic Dung Beetle *Onthophagus binodis* .” Journal of Insect Physiology 49, no. 9: 817–822. 10.1016/S0022-1910(03)00117-3. 16256683

[ece374477-bib-0026] Krüger, K. , and C. H. Scholtz . 1998a. “Changes in the Structure of Dung Insect Communities After Ivermectin Usage in a Grassland Ecosystem. I. Impact of Ivermectin Under Drought Conditions.” Acta Oecologica 19, no. 5: 425–438. 10.1016/S1146-609X(98)80048-9.

[ece374477-bib-0027] Krüger, K. , and C. H. Scholtz . 1998b. “Changes in the Structure of Dung Insect Communities After Ivermectin Usage in a Grassland Ecosystem. II. Impact of Ivermectin Under High‐Rainfall Conditions.” Acta Oecologica 19, no. 5: 439–451. 10.1016/S1146-609X(98)80049-0.

[ece374477-bib-0028] Kühne, R. 1996. “Relations Between Free‐Living Nematodes ‘and Dung‐Burying *Geotrupes* spp. (Coleoptera: Geotrupini).” Fundamental and Applied Nematology 19, no. 3: 263–271.

[ece374477-bib-0029] Lee, C. , and R. Wall . 2006a. “Distribution and Abundance of Insects Colonizing Cattle Dung in South West England.” Journal of Natural History 40, no. 17–18: 1167–1177. 10.1080/00222930600822597.

[ece374477-bib-0030] Lee, C. , and R. Wall . 2006b. “Cow‐Dung Colonization and Decomposition Following Insect Exclusion.” Bulletin of Entomological Research 96, no. 3: 315–322. 10.1079/ber2006428. 16768820

[ece374477-bib-0031] Lewis, M. J. , R. K. Didham , T. A. Evans , and J. D. Berson . 2024. “Experimental Evidence That Dung Beetles Benefit From Reduced Ivermectin in Targeted Treatment of Livestock Parasites.” Science of the Total Environment 945: 174050. 10.1016/j.scitotenv.2024.174050. 38906290

[ece374477-bib-0034] Mann, C. M. , S. Barnes , B. Offer , and R. Wall . 2015. “Lethal and Sub‐Lethal Effects of Faecal Deltamethrin Residues on Dung‐Feeding Insects.” Medical and Veterinary Entomology 29, no. 2: 189–195. 10.1111/mve.12104. 25594879

[ece374477-bib-0035] Manning, P. , S. A. Beynon , and O. T. Lewis . 2017. “Quantifying Immediate and Delayed Effects of Anthelmintic Exposure on Ecosystem Functioning Supported by a Common Dung Beetle Species.” PLoS One 12, no. 8: e0182730. 10.1371/journal.pone.0182730. PMC5553719 28800623

[ece374477-bib-0036] Microsoft , and S. Weston . 2022a. foreach: Provides Foreach Looping Construct. https://github.com/RevolutionAnalytics/foreach.

[ece374477-bib-0037] Microsoft , and S. Weston . 2022b. doParallel: Foreach Parallel Adaptor for the parallel Package. https://github.com/RevolutionAnalytics/doparallel.

[ece374477-bib-0038] O'Hea, N. M. , L. Kirwan , P. S. Giller , and J. A. Finn . 2010. “Lethal and Sub‐Lethal Effects of Ivermectin on North Temperate Dung Beetles, *Aphodius ater* and *Aphodius rufipes* (Coleoptera: Scarabaeidae).” Insect Conservation and Diversity 3, no. 1: 24–33. 10.1111/j.1752-4598.2009.00068.x.

[ece374477-bib-0039] Pokhrel, M. R. , S. C. Cairns , and N. R. Andrew . 2020. “Dung Beetle Species Introductions: When an Ecosystem Service Provider Transforms Into an Invasive Species.” PeerJ 8: e9872. 10.7717/peerj.9872. PMC7531351 33062417

[ece374477-bib-0040] R Core Team . 2025. R: A Language and Environment for Statistical Computing. https://www.R‐project.org/.

[ece374477-bib-0041] Raol, J. R. 2016. “Robot Path and Motion Planning.” In Mobile Intelligent Autonomous Systems, edited by J. R. Raol and A. K. Gopal , 151–169. CRC Press. 10.1201/b12690.

[ece374477-bib-0042] Römbke, J. , K. D. Floate , R. Jochmann , et al. 2009. “Lethal and Sublethal Toxic Effects of a Test Chemical (Ivermectin) on the Yellow Dung Fly ( *Scathophaga stercoraria* ) Based on a Standardized International Ring Test.” Environmental Toxicology and Chemistry 28, no. 10: 2117–2124. 10.1897/08-599.1. 19432504

[ece374477-bib-0043] Sánchez‐Bayo, F. , and K. A. G. Wyckhuys . 2019. “Worldwide Decline of the Entomofauna: A Review of Its Drivers.” Biological Conservation 232: 8–27. 10.1016/j.biocon.2019.01.020.

[ece374477-bib-0044] Sands, B. , and R. Wall . 2018. “Sustained Parasiticide Use in Cattle Farming Affects Dung Beetle Functional Assemblages.” Agriculture, Ecosystems and Environment 265: 226–235. 10.1016/j.agee.2018.06.012.

[ece374477-bib-0045] Tixier, T. , J. P. Lumaret , and G. T. Sullivan . 2015. “Contribution of the Timing of the Successive Waves of Insect Colonisation to Dung Removal in a Grazed Agro‐Ecosystem.” European Journal of Soil Biology 69: 88–93. 10.1016/j.ejsobi.2015.06.001.

[ece374477-bib-0046] Tonelli, M. , J. R. Verdú , F. Morelli , and M. Zunino . 2020. “Dung Beetles: Functional Identity, Not Functional Diversity, Accounts for Ecological Process Disruption Caused by the Use of Veterinary Medical Products.” Journal of Insect Conservation 24, no. 4: 643–654. 10.1007/s10841-020-00240-4.

[ece374477-bib-0047] Urrutia, M. A. , V. Cortez , R. Rosa‐García , U. García‐Prieto , and J. R. Verdú . 2024. “Analysing the Effect of Ivermectin on the Volatile Organic Compounds of Dung and Its Possible Influence on Attraction to Dung Beetles.” Ecological Entomology 49, no. 3: 386–396. 10.1111/een.13314.

[ece374477-bib-0048] Vale, G. A. , and I. F. Grant . 2002. “Modelled Impact of Insecticide‐Contaminated Dung on the Abundance and Distribution of Dung Fauna.” Bulletin of Entomological Research 92, no. 3: 251–263. 10.1079/ber2002162. 12088542

[ece374477-bib-0055] Villar, D. , and D. J. Schaeffer . 2023. “Ivermectin Use on Pastured Livestock in Colombia: Parasite Resistance and Impacts on the Dung Community.” Revista Colombiana De Ciencias Pecuarias 36, no. 1: 3–12. 10.17533/udea.rccp.v36n1a2.

[ece374477-bib-0054] Wall, R. , and S. Beynon . 2012. “Area‐Wide Impact of Macrocyclic Lactone Parasiticides in Cattle Dung.” Medical and Veterinary Entomology 26, no. 1: 1–8. 10.1111/j.1365-2915.2011.00984.x. 22007907

[ece374477-bib-0056] Wall, R. , E. Morgan , S. Timothy , and G. Sabatini . 2026. “Managing the Environmental Impact of Livestock Endectocides Through Refugia‐Based Control.” Trends in Parasitology 42, no. 6: 442–444. 10.1016/j.pt.2026.04.004. 42070959

[ece374477-bib-0049] Weaving, H. , B. Sands , and R. Wall . 2020. “Reproductive Sublethal Effects of Macrocyclic Lactones and Synthetic Pyrethroids on the Dung Beetle *Onthophagus similis* .” Bulletin of Entomological Research 110, no. 2: 195–200. 10.1017/S0007485319000567. 31571556

[ece374477-bib-0050] Wickham, H. 2016. ggplot2: Elegant Graphics for Data Analysis. R Package Version 4.0.0. Accessed March 9, 2026. https://ggplot2.tidyverse.org.

[ece374477-bib-0051] Wickham, H. 2025. stringr: Simple, Consistent Wrappers for Common String Operations. R Package Version 1.6.0. Accessed March 9, 2026. https://stringr.tidyverse.org.

[ece374477-bib-0052] Wickham, H. , R. François , L. Henry , K. Müller , and D. Vaughan . 2023. dplyr: A Grammar of Data Manipulation. R Package Version 1.1.4. Accessed March 9, 2026. https://dplyr.tidyverse.org.

[ece374477-bib-0053] Wickham, H. , D. Vaughan , and M. Girlich . 2026. tidyr: Tidy Messy Data. R Package Version 1.3.2. Accessed March 9, 2026. https://tidyr.tidyverse.org.

